# Long-term studies in cognitive training for older adults: a systematic review

**DOI:** 10.1590/1980-5764-DN-2021-0064

**Published:** 2022-04-29

**Authors:** Thais Bento Lima da Silva, Jéssica Souza Bratkauskas, Maurício Einstoss de Castro Barbosa, Guilherme Alves da Silva, Mariana Garcia Zumkeller, Luiz Carlos de Moraes, Patrícia Prata Lessa, Neide Pereira Cardoso, Tiago Nascimento Ordonez, Sonia Maria Dozzi Brucki

**Affiliations:** 1 Universidade de São Paulo, Escola de Artes, Ciências e Humanidades, São Paulo SP, Brazil.; 2 Instituto Supera de Educação, São José dos Campos SP, Brazil.; 3 Universidade de São Paulo, Faculdade de Medicina, Hospital das Clínicas, Grupo de Neurologia Cognitiva e Comportamental, São Paulo SP, Brazil.

**Keywords:** Aging, Aged, Cognition, Cognitive Aging, Time, Envelhecimento, Idoso, Cognição, Envelhecimento Cognitivo, Tempo

## Abstract

Studies show that aging is accompanied by losses in cognitive functions and that interventions can increase performance and/or support the maintenance of cognitive skills in the elderly.

**Objective:**

The objective of this study was to carry out a systematic review of long-term studies involving cognitive training (CT) in older adults without dementia and/or with mild cognitive impairment (MCI).

**Methods:**

A systematic review of controlled studies was published in scientific journals from 2000 onward, with duration ≥6 months, CT intervention, cognitively normal (CN) or MCI participants aged ≥60 years, and assessments using cognitive and/or neuropsychological tests.

**Results:**

A total of 32 studies were reviewed, comprising 10 on study protocols, 14 in CN older adults (no MCI and/or dementia), and 8 in older adults with MCI or at risk for dementia.

**Conclusions:**

The studies reported improvements in cognitive performance for some motor abilities, among older participants of CT with or without booster sessions, including multimodal interventions or otherwise.

## RESUMO.

Estudos mostram que o envelhecimento é acompanhado por perdas nas funções cognitivas, e que as intervenções podem gerar um aumento no desempenho e/ou apoiar a manutenção de habilidades cognitivas em idosos.

**Objetivo:**

Realizar uma revisão sistemática de pesquisas com longa duração que ofereceram treino cognitivo (CT) em idosos sem demência e/ou com comprometimento cognitivo leve (MCI).

**Métodos:**

Revisão sistemática de estudos controlados, publicados em periódicos científicos a partir de 2000, com duração ≥6 meses, CT na intervenção, participantes ≥60 anos, saudáveis ou com MCI, avaliações por testes cognitivos e/ou neuropsicológicos.

**Resultados:**

Foram selecionados 32 estudos, sendo dez protocolos de pesquisa, 14 com idosos sem MCI e/ou sem demência e oito com idosos com MCI ou risco de demência.

**Conclusões:**

Foram relatados benefícios no desempenho cognitivo, incluindo habilidades motoras, de idosos que participaram de CT, com ou sem sessões de reforço, incluindo ou não intervenções multimodais.

## INTRODUCTION

According to the estimates of the United Nations 2019 Revision of World Population Prospects^1^, 17.8% of people in the world will be above age 65 by 2060, up from 9.6% in 2021. In Brazil, this proportion will increase from 9.9 to 27%. During the normal cognitive aging process, the organism undergoes periods of stability and change. These changes partially stem from physiological and anatomical components. Most notable of the normal changes accompanying healthy aging are the aspects relating to the brain and cognitive functioning^2^, which may affect more complex everyday tasks, such as driving, paying bills, and remembering dates and appointments^3^.

Studies show that aging is accompanied by losses in cognitive functions and that interventions can promote performance gains and/or support the maintenance of cognitive abilities in healthy older persons^3,4^. According to the literature, the existing cognitive interventions, such as cognitive training (CT), have the potential for promoting health by optimizing cognitive and neural plasticity. These effects may be increased by combining CT with other types of interventions, such as physical activities and a balanced diet, with the aim of improving the performance of the individual and neural reorganization as a result of the intervention^5,6^. Both neural and cognitive plasticity are an inherent part of the life course of an individual. Although diminishing with age, plasticity supports the learning of mnemonic techniques, as well as the expansion and integration of knowledge related to cognitive functions^7^.

The effects of CT can extend to other domains, such as health promotion^8,9^, as well as functioning^10^. A plethora of cognitive modalities have been tested in both healthy and cognitively impaired elderly, displaying similar positive effects on cognitive performance and other variables, such as psychological well-being. According to Ngandu et al.^6^, intervention might not be too late for presymptomatic and predementia disease stages and also for at-risk states, such as in mild cognitive impairment (MCI). For example, recent studies by Peng et al.^11^, Valdés et al.^12^, Lee et al.^13^, and Djabelkhir et al.^14^. reported cognitive gains in older adults with MCI. The research by Lee et al.^13^ indicated benefits even for mild dementia.

One of the first CT studies of the long-term type, i.e., lasting 6 consecutive months or longer, was the multicenter study conducted by Ball et al.^15^, involving a large number of participants: 2,832 subjects aged 65–94 years. Participants received one of the following types of cognitive intervention: (a) verbal episodic memory training (group 1), (b) logical reasoning training (group 2), (c) processing speed training (group 3), or (d) control group without training (group 4). A total of 10 training sessions were given and 60% of the sample received 4 booster sessions after 11 months. The results showed that 87% of participants from group 3 improved performance on processing speed, 74% from group 2 improved logical reasoning, and 26% from group 1 improved memory. Booster sessions were effective for maintaining processing speed and logical reasoning, but not for episodic memory. The positive effects of training were not detected during the daily routine of participants, but persisted after 2 years, suggesting that the intervention effects can remain consistent over the long term.

Ngandu et al.^6^, in a study involving a multidomain intervention of nutritional diet, physical exercise, CT, and vascular risk monitoring, reported this to be more effective and efficient, showing 20–150% of improvement in cognitive performance of participants. The Finnish Geriatric Intervention Study to Prevent Cognitive Impairment and Disability (FINGER) described the effects of this approach, showing that the intensity of the intervention, the target public, the type of approach, and the fact of being long-term training explained the beneficial effects for cognition observed in participants. These results reveal the importance of healthy dietary habits and regular physical exercise in conjunction with cognitive interventions.

Training studies differ not only in duration but also in strategies trained and methodology employed. Results reported in the literature varied widely regarding the strength of effects, generalization to untrained tasks, and long-term maintenance of improvements^16^. This heterogeneity justifies an analysis of the literature on long-term cognitive interventions with the aim of adding to the knowledge on CT and providing consistent, in-depth information on the cognitive benefits associated with long-term interventions and the strategies they employ. The objective of this study was to carry out a systematic review of long-term studies involving CT in older adults without dementia and/or with MCI.

## METHODS

This systematic review had its protocol registered in the International Prospective Register of Systematic Reviews (PROSPERO) in April 2021 (submitted in February 2021) under registration number CRD42021239130. The protocol can be assessed at https://www.crd.york.ac.uk/prospero/display_record.php?ID=CRD42021239130.

Eligibility criteria were as follows: clinical trial studies with a 6-month duration or longer, intervention involving CT and a control group, cognitively normal (CN) or MCI participants aged ≥60 years, articles published in 2000 or later in scientific journals, and follow-up assessments of intervention effects using cognitive and/or neuropsychological tests. The criterion used for CT studies to be considered long term was to have a duration of 6 consecutive months or more.

Publications of master’s dissertations, book chapters, doctoral theses, letters to the editor and case studies, studies whose samples included individuals aged <60 years or with dementia, studies performed at long-term care institutions, and studies failing to report the effects of intervention on cognitive performance were excluded.

The systematic review was conducted between February and April 2021. All manuscripts in Portuguese and English were revised for eligibility criteria. The Scielo, LILACS, and PubMed/MEDLINE scientific databases were searched using the following combinations of the key words: ((idoso OR idosos OR idosa OR idosas) OR (elder OR “older person” OR “older persons” OR “older people” OR “senior citizen” OR “senior citizens” OR elderly OR “aging people” OR “aging person” OR “aging persons”)) AND (“treino cognitivo” OR “cognitive training”) AND (“longa duração” OR “long term” OR longitudinal OR “follow up”) AND (envelhecimento OR aging).

To guide the stages of identification, screening, and eligibility of studies, two pairs of reviewers working independently screened all records retrieved, following the steps of the Statement of Preferred Reporting Items for Systematic Reviews and Meta-Analyses (PRISMA)^17^. The initial identification of studies was performed by searching the abovementioned databases. In the screening stage, duplicate studies were excluded, and titles and abstracts were read for the first selection, according to the preestablished inclusion and exclusion criteria. In the eligibility stage, the remaining studies were read in full in order to be selected according to the same criteria. The remaining studies after this stage were the studies included in the review.

The following data were extracted from the articles: study title, authors’ name, summary, results, methods, justification, objectives, and conclusion. Excel spreadsheets were used as support tools in this process.

The studies were assessed for quality according to Downs and Black’s^18^ checklist. This assessment tool consists of 27 questions, which are divided into 5 subscales: report or assessment of adequate information (10 items), external validity (3 items), internal validity of detailed measurements and result bias (7 items), confounding factors (6 items), and power (1 item). Each item that makes up the checklist assigns a score from 0 to 1, except for the item that assesses the description of confounding factors, which can assign up to 2 points, and the item that assesses the description of the study’s power (27), which originally assigned from 0 to 5 points, but was modified to assign from 0 to 1 point, as in other studies^19–21^ so that a score of 1 was given if the article presented power calculation and/or sample size calculation and a score of 0 if it did not present any of these calculations. Thus, the checklist has total scores ranging from 0 to 28 points. For a better understanding of the data obtained, the score was converted into a percentage for each domain, and a final average of the total score of all domains was calculated. Next, the quality of the articles was classified as follows: up to 0.39 was considered bad, 0.40–0.69 considered regular, 0.70–0.79 considered good, and 0.80 or above was considered excellent.

## RESULTS

**Figure 1:**
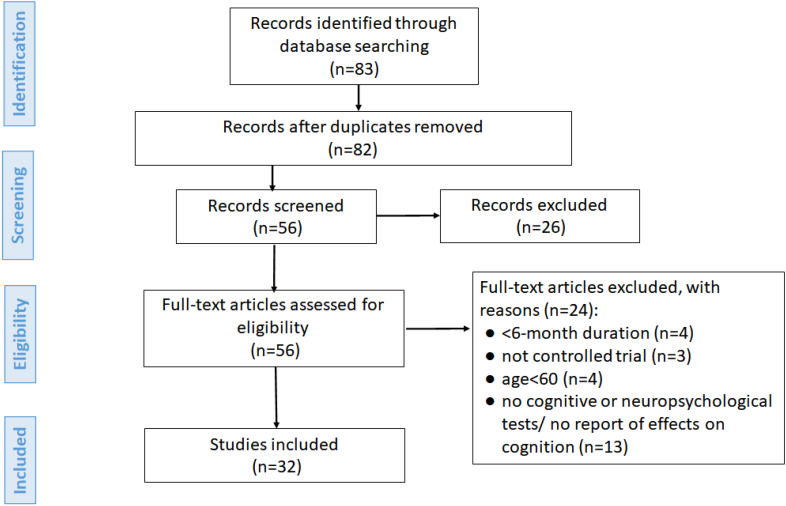
Flowchart showing study selection process.^17^

The initial search led to the retrieval of 83 studies, of which 1 was subsequently excluded owing to duplication. Titles and abstracts of the remaining 82 studies were read and screened for relevance to the review topic. After applying inclusion and exclusion criteria, a total of 27 studies were excluded. Thus, 56 articles were read in full; of these, 24 studies that did not meet the eligibility criteria were excluded. The process of study selection for inclusion in the review is shown in Figure 1. The final 32 studies included in the review for analysis are listed in Tables 1–3.

**Table 1:** Long-term studies with cognitively normal older adults.

Authors	Sample	Objectives	Main intervention	Results found	Downs and Black
Willis et al.^41^	n=2,832 (mean age, 73.6 years)	To determine the effects of cognitive training on daily function and durability of training on cognitive abilities.	Ten-session training for memory (verbal episodic memory), reasoning (inductive reasoning), or speed of processing (visual search and identification); four-session booster training at 11 and 35 months after training in a random sample of those who completed training.	Reasoning training resulted in less functional decline in self-reported IADL. CT cognitive training improved cognitive abilities specific to the abilities trained that continued 5 years after initiation of intervention.	24
Gross and Rebok^40^	n=1,401 (mean age, 73.8 years)	To report long-term impact of memory training on strategy use and longitudinal associations between strategy clustering, memory performance, and everyday functioning.	Data from the Advanced Cognitive Training for Independent and Vital Elderly (ACTIVE) study (n=1,401) were used to describe strategy use in a community-dwelling sample of older adults. Strategy clustering scores on verbal list learning tasks of episodic memory were used to test the impact of memory training on strategy use and study longitudinal associations between strategy clustering, memory performance, and everyday functioning.	Memory training improved strategy use. Effects were maintained for up to 5 years. The strategies were positively associated with memory performance and everyday functioning.	24
Ball et al.^43^	n=2,802 (mean age, 73.6 years)	To examine the longitudinal impact of dosage (number of training sessions) on improvement and maintenance of cognitive abilities and everyday functions.	Participants were randomly assigned to one of four groups: 10-session group training for memory (verbal episodic memory; n=711), or reasoning (ability to solve problems that follow a serial pattern; n=705), or speed of processing (visual search and identification; n=712); or a no-contact control group (n=704). For the three treatment groups, four-session booster training was offered to a 60% random sample 11 months later.	Initial SOPT effects were maintained over 5 years and amplified by booster sessions. A single booster session counteracted 4.92 months of age-related processing speed decline.	24
Borella et al.^36^	n=36 (above 75 years of age)	To examine whether WM training can improve WM performance in old-old individuals and produce and maintain transfer effects on untrained tasks.	2 weeks, 60 min per session, memory training (n=18), active control (n=18); assessments: pre and post-test; follow-up: after 8 months; tests: CWMS task, Dot Matrix, Forward and Backward Digit Span, Cattell, pattern comparison task, and Stroop Color task.	The WM training program produced benefits maintained over time even in old-old adults, confirming there is still room for plasticity in the basic mechanisms of cognition in advanced old age.	22
Gross et al.^37^	n=1,401 (mean age, 73.8 years)	To investigate the influence of memory training on initial recall and learning.	Each ACTIVE intervention was administered in 10 small-group training sessions, each lasting 60–75 min, offered over a course of 10 weeks. The first of 10 sessions provided didactic training on how memory works and how to maximize benefits of training.	Memory strategy training was associated with significant long-term gains in learning, stemming from both the highly significant effect of the training and from a slower decline, for up to 5 years, in memory span.	24
Jones et al.^42^	n=1,659 (mean age, 73.7 years)	To determine the influence of CT in the ACTIVE study on the pace of cognitive aging.	Briefly, older adults (aged 65–94) were randomly assigned to one of the three cognitive training or no contact control arms. Training lasted 5–6 weeks, and participants were assessed pre- and post-intervention, and at 1, 2, 3, 5, and 10 years after post-test. This analysis considers outcomes through 5 years, as the 10-year main results are currently under analysis.	Reasoning training attenuated aging-related training. Memory gains were maintained but about half of reasoning and speed gains were lost. All trained groups performed better than controls at 5 years. Performance differences at end of follow-up were equivalent to about 6, 4, and 8 years of aging for memory, reasoning, and speed training, respectively.	24
Kwok et al.^33^	n=223 (mean age, 75.4 years)	To examine the short- and long-term effects of a cognitive training (CT) program in enhancing cognitive function of older people with subjective memory complaints.	A single-blind randomized placebo-controlled trial was carried out in a sample of 223 older adults aged 65 years or above with subjective memory complaints in Hong Kong. They were randomly assigned to either receive CT (intervention group, n=111) or attend health-related educational lectures only (control group, n=112). Participants’ cognitive abilities were assessed by the Chinese version of Mattis Dementia Rating Scale at baseline, immediately after the training, and 9 months after the training.	Cognitive training was effective in enhancing the overall cognitive functioning of less educated older adults with subjective memory complaints. The positive effect was durable for at least 9 months in conceptualization and memory.	23
Sisco et al.^39^	n=1,912 (mean age, 72.9 years)	To investigate how a multicomponent memory intervention affected memory for prose.	Participants were randomized into one of the three training arms (i.e., memory, reasoning, and speed of processing) or a no-contact control group; about half of the trained participants received additional booster training 1 and 3 years post intervention.	Multi-factorial memory training can improve verbatim recall for prose, but the effect does not last without continued intervention.	24
Gross et al.^44^	n=1,401 (mean age, 73.3 years)	To evaluate whether training can increase the use of MoL and whether MoL is associated with better memory maintained over time.	The authors analyzed skip patterns on response forms for the Auditory Verbal Learning Test (AVLT) in the Advanced Cognitive Training for Independent and Vital Elderly (ACTIVE; n=1,401) trial using 5 years of longitudinal follow-up.	The use of MoL was associated with improved memory sustained over time. Changes in strategies resulted in differences in memory performance.	25
Linde et al.^35^	n=70 (mean age, 66.8 years)	To analyze the short- and long-term effects of PT, combined CT, and PT plus CT programs on age-sensitive fluid cognitive abilities.	70 healthy senior citizens (age 60–75) were allocated to a physical, cognitive, combined physical plus cognitive, and waiting control group. The trial assessed information processing speed, short-term memory, spatial relations, concentration, reasoning, and cognitive speed.	Physical, cognitive, and combined physical plus cognitive activity can be seen as cognition-enrichment behaviors in healthy older adults that show different rather than equal intervention effects.	21
Rebok et al.^10^	n=2,832 (mean age,73.6 years)	To determine the effects of cognitive training on cognitive abilities and everyday function over 10 years.	Ten training sessions for memory, reasoning, or speed of processing; four sessions of booster training 11 and 35 months after initial training.	Ten training sessions for memory, reasoning, or speed of processing, four sessions of booster training 11 and 35 months after initial training tests; tests: RAVLT, HVLT, RBPR, Letter Series, Letter Sets, Word Series, UFOV, MDS-HC, EPT, OTDL, CRT, and TIADL.	24
Eggenberger et al.^32^	n=89 (mean age, 78.9 years)	To evaluate synergistic effects of multicomponent PT complemented with novel simultaneous CT on cognition in older adults.	Seniors, older than 70 years, without cognitive impairment, were randomly assigned to either: (1) virtual reality video game dancing (DANCE), (2) treadmill walking with simultaneous verbal memory training (MEMORY), or (3) treadmill walking (PHYS). Each program was complemented with strength and balance exercises. Two 1-h training sessions per week over 6 months were applied.	Particular executive functions benefit from simultaneous cognitive–physical training compared to exclusively physical multicomponent training. Cognitive–physical training programs may counteract widespread cognitive impairments in the elderly.	22
Li et al.^34^	n=270 (mean age, 69.8 years)	To examine the relationship between changes in spontaneous brain activity and cognitive performance that occur after CT.	Participants were trained for 1 h, twice a week, for 12 weeks. Cognition was assessed in all participants and magnetic resonance images were obtained at baseline and 1 year after training. To assess spontaneous fluctuations in brain activity, we acquired resting-state fMRI data. Two indices—functional entropy and time-domain entropy—were used to measure the effects of training. Functional entropy increases with aging and indicates disruptions in functional connectivity. Time-domain entropy decreases with aging and indicates structural alterations in the brain and blood-flow reduction.	Seventy participants completed the study: 26 in the multidomain cognitive training group (70.38±3.30 years), 27 in single-domain group (70.48±3.93 years), and 17 in a control group (68.59±3.24 years). Functional entropy increased significantly less in the multi-domain (p=0.047) and single-domain groups (p=9.51×10^−4^) compared with the control group. In the multi-domain group, this was true in the paracentral lobule (p=0.004, Bonferroni corrected p<0.05). Time-domain entropy also improved with training. Compared with controls, time-domain entropy in the multi-domain group decreased less in the inferior frontal gyrus pars opercularis (p=3.59×10^−4^), the medial part of superior frontal gyrus (p=1.17×10^−5^), and the thalamus (p=4.72×10^−5^), while that in the single-domain group decreased less in the cuneus (p=2.58×10^−4^, Bonferroni corrected p<0.05).	24
Ross et al.^38^	n=2,802 (mean age, 73.6 years)	To assess the impact of three CT programs on objective measures of physical functioning across 5 years.	Older adults randomized into a processing speed (n=702), reasoning (n=694), or memory (n=703) training intervention were compared to those randomized into a no-contact control condition (n=698). Intention-to-treat (ITT) and treatment-received (time-varying number of training sessions) analyses were conducted.	There were no transfer effects in the ITT analyses. Treatment-received models demonstrated that training sessions (i.e., higher dosage) across all intervention arms transferred to better maintained Digit Symbol Copy and Turn 360 performance relative to the control group. More reasoning training transferred to better grip strength.	23

CN: cognitively normal controls; CT: cognitive training; ACTIVE: Advanced Cognitive Training for Independent and Vital Elderly; SOPT: speed of processing training; HVLT: Hopkins Verbal Learning Test; AVLT: Rey Auditory-Verbal Learning Test; RBPR: Rivermead Behavioral Paragraph Recall; EPT: everyday problems test; IADL: instrumental activities of daily living; OTDL: observed tasks of daily living; TIADL: timed instrumental activities of daily living; CRT: complex reaction time test; CES-D: Center for Epidemiological Studies–Depression scale; MMSE: Mini-Mental State Examination; SF-36: Short Form 36-Item; RBMT: Rivermead Behavioral Memory Test; PT: physical training; UFOV: useful field of view; CWMS: categorization working memory span; RAVLT: Rey Auditory-Verbal Learning Test; CMSS: Chinese Memory Symptoms Scale; CMMSE: Chinese version of Mini-Mental State Examination; WM: working memory; CDRS: Chinese version of Mattis Dementia Rating Scale; MoL: method of loci; LPS: LeistungsPrüfSystem; TMT-A: Trail-Making Test Part A; MDS-HC: Minimum Dataset – Home Care, TMT-B: Trail-Making Test Part B, PAL: paired-associates learning; WMS-R: Wechsler Memory Scale – Revised; WAIS-R: Wechsler Adult Intelligence Scale – Revised; DSST: Digit Symbol Substitution Test; PACES: Physical Activity Enjoyment Scale; RBANS: Repeatable Battery for the Assessment of Neuropsychological Status (Form A); CWST: Color Word Stroop Test; DSC: Digit Symbol Copy.

**Table 2:** Long-term studies with older adults with MCI or risk for dementia.

Authors	Sample	Objectives	Main intervention	Results found	Downs and Black
Rozzini et al.^49^	n=59 (between 63 and 78 years of age)	To evaluate the efficacy of an NPT in patients with MCI treated with ChEIs, compared with patients MCI treated only with ChEIs, in a longitudinal, one year follow-up study.	ChEIs, ChEIs+NPT, control; assessments: pre-test; follow-up 3 months after intervention; tests: MMSE, category fluency and letter fluency, Raven’s colored matrices, Rey’s figure – delayed recall and copy, NPI-Q, GDS, BADL, and IADL.	Subjects treated with TNP+ChEIs showed improvements in episodic memory, abstract reasoning and behavioral disturbances, long-term NPT in ChEIs-treated MCI subjects induces additional cognitive and mood benefits.	21
Valdes et al.^51^	n=2,802 (mean age, 77.6 years)	To examine the longitudinal effects of SOPT among older adults with psychometrically defined MCI from the ACTIVE trial	SOPT (n=702), reasoning training (n=694), memory training (n=703), control (n=698); booster: Participants completed eight or more training sessions, four sessions before assessments at years 1 and 3, training and control groups; assessments: pre-test and post-test (2 months later); follow-up: 1, 2, 3, and 5 years after pre-test.	Immediate improvement in participants with MCI, particularly the non-amnestic subtype. Initial training gains were maintained, where all subtypes showed similar trajectories across 5 years, with no significant changes in performance. SOPT proved effective and promoted durable effects.	24
Rojas et al.^48^	n=46 (mean age, 76.5 years)	To examine the efficacy of a CIP in patients with MCI and to assess patients’ condition at 1-year follow-up.	CT (n=24), control (n=22); assessments: pre-test; follow-up: 1 year; tests: MMSE, CDR, Signoret’s memory battery, BNT, verbal fluency, WASI-II, TMT-A, WAIS-III, TMT-B, QoL questionnaire, NPI, and the IADL scale.	Persons with MCI can improve their performance on cognitive and functional measures, and effects could persist in the long term. CT in MCI may prevent cognitive decline or slow conversion to dementia.	20
Law et al.^50^	n=83 (mean age, 73.6 years)	The aim of this study was to compare the effects of a functional tasks exercise program to a cognitive training program in older adults with mild cognitive impairment.	Participants were randomized into either a functional task exercise group (n=43) or an active cognitive training group (n=40) for 10 weeks. All outcome measures were undertaken at baseline, post-intervention, and 6-month follow-up using Neurobehavioral Cognitive Status Examination, Trail Making Test, Chinese Version Verbal Learning Test, Category Verbal Learning Test, Lawton Instrumental Activities of Daily Living Scale, and Problems in Everyday Living Test.	The FcTSim promoted significant sustained improvements in general cognitive functions, executive function, and problem-solving ability, as well as promoting brain plasticity.	22
Ngandu et al.^6^	n=1,260 (mean age, 69.4 years)	To assess a 2-year multidomain intervention in elderly people from the general population at risk for cognitive problems.	Multi-domain intervention (n=631), control (n=629); assessments: pre-test, post-test; follow-up: 1, 2, and 7 years after intervention; tests: NTB, Zung scale, SPPB, and CAIDE.	Results suggested a multi-domain intervention could improve or maintain cognitive functioning in elderly people at risk for cognitive problems.	25
Bahar-Fuchs et al.^46^	n=44 (mean age, 74.6 years)	To evaluate the extent to which CCT benefits older adults with MCI and MrNPS and examine its effects on meta-cognitive and non-cognitive outcomes.	CCT (n=21), active control (n=23); assessments: pre and post-test; follow-up: 3 months; tests: NIA-AA, BADL, NPI-Q, and ANZCTR.	Home-based CCT with adaptive difficulty and personal tailoring appears superior to more generic CCT in relation to both cognitive and non-cognitive outcomes.	23
Zhao et al.^45^	n=93 (mean age, 70.1 years)	To explore the effects of a CrExp program on cognitive functioning in older adults with MCI.	CrExp (n=48), control (n=45); assessments: pre and post-test; follow-up: 6 months; tests: MoCA, CVAVLT, CVCVFT, DST, TMT-A, TMT-B, CVADL, and MSQ.	CrExp therapy has greater positive effects on cognitive functions and daily living ability than standard cognitive training. This unique therapy may serve as a cost-effective adjunct to standard interventions for older adults with mild cognitive impairment.	22
Belleville et al.^47^	n=145 (mean age, 72.2 years)	To assess the effect of memory training on the cognitive functioning of persons with MCI and its durability and to evaluate whether this effect generalizes to daily life and whether positive effects can be obtained from psychosocial intervention.	Memory training (n=49), psychosocial intervention (n=49), control (n=47); booster intervention: one session after assessment at 3 months; assessments: pre and post-test; follow-up: 3 and 6 months; tests: GAI, GDS, GWBS, MMQ, QAM, ADL-PI, Free and Cued Recall memory test, EPI, EPR, Inventaire d’Activities Physiques, and GSE.	CT group showed an improvement on delayed memory and use of strategy use in everyday life, maintained at follow-up. Participants in psychosocial intervention group did not show any significant improvement.	24

NPT: neuropsychological training; MCI: mild cognitive impairment; ChEIs: cholinesterase inhibitors; MMSE: Mini-Mental State Examination; NPI-Q: Neuropsychiatric Inventory Questionnaire; GDS: Geriatric Depression Scale; BADL: Bristol Activities of Daily Living Scale; IADL: instrumental activities of daily living; SOPT: speed of processing training; ACTIVE: Advanced Cognitive Training for Independent and Vital Elderly; HVLT: Hopkins Verbal Learning Test; RAVLT: Rey Auditory-Verbal Learning Test; RBMT: Rivermead Behavioral Memory Test; UFOV: useful field of view; CIP: cognitive intervention program; CDR: clinical dementia rating; BNT: Boston Naming Test; WASI-II: Vocabulary from the Wechsler Abbreviated Scale of Intelligence – Block Design; TMT-A: Trail-Making Test A; WAIS-III: Wechsler Adult Intelligence Scale III; FcTSim: simulated functional tasks; TMT-B: Trail-Making Test B; CVAVLT: Chinese Version of the Auditory Verbal Learning Test; CVCVFT: Chinese Version of the Category Verbal Fluency Test; NTB: neuropsychological test battery; SPPB: Short Physical Performance Battery; CAIDE: Cardiovascular risk factors, aging and dementia; NIA-AA: National Institute on Aging – Alzheimer’s Association; NPI-Q: Neuropsychiatric Inventory Questionnaire; ANZCTR: Australian-New-Zealand Clinical Trial Registry; CrExp: creative expression; MoCA: Montreal Cognitive Assessment; DST: Digit Span Test; CVADL: Chinese Version of Activities of Daily Living Scale; MSQ: Memory Satisfaction Questionnaire; GAI: Geriatric Anxiety Inventory; GWBS: General Well-Being Schedule; MMQ: Multifactorial Memory Questionnaire – Memory Strategies; QAM: Questionnaire d’Auto-Evaluation de la Mémoire; ADL-PI: Activities of Daily Living – Prevention Instrument questionnaire; EPI: Eysenck Personality Inventory; EPR: Echelle de Préférence de Routinisation; GSE: General Self-Efficacy Scale; MrNPS: Mood-related neuropsychiatric symptoms; CCT: computerized cognitive training.

**Table 3:** Publications of study protocols describing methods and planning.

Authors	Sample characteristics	Objectives	Main intervention	Results	Downs and Black
Jobe et al.^31^	n=2,832 (mean age, 73.6 years)	To determine the effects of three different CIP on improvement in performance of cognitively based measures under laboratory or field conditions and on measures of cognitively demanding everyday functioning associated with independent living.	SOPT, reasoning training, memory training, control; booster intervention: Participants shall complete eight training sessions or more, 11 months after the end of the primary training, 4 sessions, 3 weeks; assessments: pre- and post-test; follow-up: 12 and 24 months after pre-test; tests: MMSE, RAVLT, HVLT, RBMT, TIADL, Related Word Lists, RBMT, RBPR, UFOV, Word Series, Letter series, Letter Sets, DSST, DSC, EPT, OTDL, CRT, MDS-HC, SF-36, Turn 360, Grip Strength, and CES-D.	Primary outcomes focus on measures of cognitively demanding everyday functioning, including financial management, food preparation, medication use, and driving. Secondary outcomes include health-related quality of life, mobility, and health-service utilization.	24
Kivipelto et al.^30^	n=1,200 (25–74 years)	To investigate to what extent a multidomain intervention can prevent/delay cognitive impairment in elderly with an elevated risk of MCI.	Nutritional guidance, PT, CT (2 6-month periods, 3 times/week, 10–15 min/session, 72 training sessions/period), social activity, intensive monitoring, and management of metabolic and vascular risk factors, control group (regular health advice). Assessments: pre-test, 1 year after pre-test and post-test; tests: mNTB, CWST, and TMT (A and B).	All 1,200 persons are enrolled and the intervention is ongoing as planned. Baseline clinical characteristics indicate that several vascular risk factors and unhealthy lifestyle-related factors are present, creating a window of opportunity for prevention. The intervention completed during 2014.	25
Lee et al.^25^	n=80 (age ≥60 years)	To determine whether combined therapies, sequential, or simultaneous are a feasible approach for training older individuals with MCI and whether they can induce superior results compared with a single intervention mode and to compare which approach is best for cognitive functions, physical fitness, ADL, and QoL.	CT, PT, sequential training, or dual-task training. Assessments: pre- and post-test; follow-up: 6 months; tests: MoCA, Stroop test, WAIS, WMS, 10-m Walk Test, BBT, TUG, CST, IPAQ, ActiGraph GX3, DAD, BI, IADL, QoLAD, CBI, GDS, and CIQ.	The results of this proposed study provide important information regarding the feasibility and intervention effects of combining physical exercise and cognitive training for older individuals with MCI.	24
Woods et al.^24^	n=360 (age ≥65 years)	To examine whether tDCS of frontal cortices enhances neurocognitive outcomes achieved from cognitive training in older adults experiencing age-related cognitive decline: the Augmenting Cognitive Training in Older Adults study (ACT).	CT+tDCS, CT+placebo, training control+tDCS, training control+placebo; assessments: Initial pre-training, after 12 weeks of CT/training control+stimulation/simulation; follow-up: 1 year after training; tests: NIH Toolbox Cognitive Function Battery, neuroimaging, SF-36, AUDIT-10, DAST-10, 10-m walk test, Beck Depression Inventory-II, State Trait Anxiety Inventory, Starkstein Apathy Scale, UCLA Loneliness Scale, Lubben Social Network Scale, Pittsburgh Sleep Quality Index, and Graded Chronic Pain Scale.	The findings from this study have the potential to significantly enhance efforts to ameliorate cognitive aging and slow dementia.	25
Montero-Odasso et al.^23^	n=200 (age ≥60 years)	To ascertain whether combined AE and RT have better effect on cognition that a BAT intervention in older adults with MCI.	(1) AE and RT+CT+vitamin D, (2) AE and RT+CT+placebo D, (3) control AE and RT+CT+vitamin D, (4) Control AE and RT+CT+placebo D, (5) control BAT+CT+placebo D; assessments: pre-test and post-test (6 months after pre-test); follow-up: 1 year; tests: ADAS-Cog 13, ADAS-Cog plus, MRI, TMT-A, TMT-B, DSST, Digit Span forward & backward, and Category Fluency, MoCA, Color Word Interference Test, 6-MWT, SPPB, SF-36, IADL, CDR, GDS-30, and GAD-7.	The SYNERGIC Trial established the efficacy and feasibility of a multimodal intervention to improve cognitive performance and mobility outcomes in MCI.	26
Sipilä et al.^22^	n=314 (70–85 years old)	To determine whether a combination of PT and CT has greater effects on walking speed, dual-task cost in walking speed, fall incidence, and executive functions compared to PT alone.	(1) PT, (2) PT+CT; assessments: pre-test; follow-up: 6 and 12 months after; tests: Stroop Test, TMT-A, TMT-B, CERAD, and Letter Verbal Fluency Test.	When completed, this study will provide new knowledge on the effects of physical and cognitive training on the prevention of walking limitations and rate of falls in older people. The expected results will be of value in informing strategies designed to promote safe walking among older people and may have a significant health and socioeconomic impact.	22
Ten Brinke et al.^28^	n=379 (65–85 years old)	To examine the effect of a CCT program, alone and preceded by a brisk walk, on cognitive function and explore the underlying neural mechanism in community – dwelling older adults.	Eight weeks sessions, three times week for 1 h+3 three times 1-h session at home; study groups: (1) computerized (FBT), (2) exercise plus CCT (Ex-FBT), and (3) active control (BAT). Assessments: pre-test and post-intervention (8 weeks); follow-up: after 1 year; tests: MoCA, MMSE, IADL, FCI, RAVLT; Toolbox Cognition Battery, Stroop Color-Word Test, TMT-A, TMT-B, DSST, SPPB; 6-MWT, PASE, and Neuroimaging.	If results from this study show benefits for cognition at trial completion, CCT programs, alone or in combination with walking, might be a strategy to promote healthy cognitive aging in older adults. In addition, results from the 1-year follow-up measurement could provide important information regarding the long-term benefits of these CCT programs.	22
VanVleet et al.^29^	n=120 (age≥65 years)	To test the effectiveness of a longer computer-based version of the TAPAT for improving cognitive abilities, functional status, and QoL in individuals with cognitive decline.	TAPAT (versions 1 and 2) (n=60), active control (n=60); evaluations: pre-test, halfway through the intervention, post-test; follow-up: after 3 months; tests: TMT-B, DKEFS Verbal Fluency, Auditory Consonant Trigrams, WAIS Digit Span, Attention Blink Task, Category Change Task, Gradual Start Continuous Performance Task, Stop Signal Task, flanker task, Stroop cross-modal, WAIS IV Digit Span, WM task, Reinforcement Learning Task, WMS IV Logical Memory I and II immediate and late recall, measurement of walking behavior, self-efficacy assessment, Fall Effectiveness Scale, TUG, SF- 12, Cognitive Failure Questionnaire, Pittsburg Sleep Quality Index, MAAS, and Breath Counting Task.	The strengths of this protocol are that it tests an innovative, in-home administered treatment that targets a fundamental deficit in adults with age-related cognitive decline; employs highly sensitive computer-based assessments of cognition as well as functional abilities, and incorporates a large sample size in an RCT design.	24
Zülke et al.^26^	n=1,152 (60–77 years)	To evaluate the effectiveness of a multi-component intervention in preventing or delaying cognitive decline in older adults at risk for dementia and to assess the effects of the intervention on mortality, nursing home placement, functioning in everyday activities, QoL, depressive symptoms, social inclusion, and cost-effectiveness of the intervention.	Compared to previous trials, AgeWell.de covers an even broader set of interventions suggested to be beneficial for the intended outcomes. The findings will add substantial knowledge on modifiable lifestyle factors to prevent or delay cognitive decline. (1) nutritional counseling, PT, CT, optimization of medication, management of vascular risk factors, social activity, and further interventions targeting grief and depression; (2) control; follow-up: 2 years; tests: TMT A and B, Word List Memorization – CERAD subtest, Verbal Fluency Test – Animals – CERAD subtest, Constructional Praxis – CERAD subtest, Reading the Mind in the Eyes Test – revised version, and MoCA.	Compared to previous trials, AgeWell.de covers an even broader set of interventions suggested to be beneficial for the intended outcomes. The findings will add substantial knowledge on modifiable lifestyle factors to prevent or delay cognitive decline.	25
Yoon et al.^27^	n=230 (mean age, 72.0 years)	To compare the effect of broad and directed (narrow) technology-based training on basic perceptual and cognitive abilities in older adults and on the performance of simulated tasks of daily living including driving and fraud avoidance.	Web-based brain game suite (Brain HQ) and strategy video game (Rise of Nations) or to directed training for IADL training using web-based programs for both driving and fraud avoidance training, active control; assessments: pre- and post-test; follow-up: 1 year after training; tests: ability tests of IADL (driving simulator test for hazard perception, and a financial fraud recognition test), UFOV, DSST, RAPM, Letter sets, HVLT, RAVLT, and UMCFAB.	The baseline results support that randomization was successful across the intervention conditions.	23

CIP: cognitive intervention program; ACTIVE: Advanced Cognitive Training for Independent and Vital Elderly; SOPT: speed of processing training; MMSE: Mini-Mental State Examination; RAVLT: Rey Auditory Verbal Learning Test; HVLT: Hopkins Verbal Learning Test; RBMT: Rivermead Behavioral Memory Test; TIADL: timed instrumental activities of daily living; RBPR: Rivermead Behavioral Paragraph Recall; UFOV: useful field of view; DSST: Digit Symbol Substitution Test; DSC: Digit Symbols Copy; EPT: everyday problems test; OTDL: observed tasks of daily living; CRT: complex reaction time; MDHC: Minimum Dataset – Home Care; SF-36: Short Form 36-Item; CES-D: Center for Epidemiological Studies – Depression scale; PT: physical training; CT: cognitive training; mNTB: modified neuropsychological test battery; CWST: Color Word Stroop Test; TMT-A: Trail-Making Test A; TMT-B: Trail-Making Test B; ADLs: activities of daily living; MoCA: Montreal Cognitive Assessment; WAIS: Wechsler Adult Intelligence Scale; WM: working memory; WMS: Wechsler Memory Scale; BBT: Box and Block Test; TUG: Timed Up and Go; CST: 30-s Chair-Stand Test; IPAQ: International Physical Activity Questionnaires; DAD: Disability Assessment for Dementia; BI: Barthel Index; IADL: instrumental activities of daily living; QoLAD: quality of life in Alzheimer’s disease instrument; CBI: caregiver burden inventory; GDS: Geriatric Depression Scale; CIQ: Community Integration Questionnaire; tDCS: transcranial Direct Current Stimulation; AUDIT-10: Alcohol Use Disorders Test; DAST-10: Drug Abuse Screening Test; AE: aerobic exercise; RT: progressive resistance training; BAT: balance and toning control; MRI: magnetic resonance imaging (Neuroimaging); 6-MWT: Six-Minute Walk Test; SPPB: Short Physical Performance Battery; CDR: clinical dementia rating; GAD-7: Generalized Anxiety Disorder 7; CERAD: Consortium to Establish a Registry for Alzheimer’s Disease; FCI: Functional Comorbidity Index; PASE: Physical Activity Scale for the Elderly; TAPAT: Tonic and Phasic Alertness Training; SF-12: Short-Form 12; MAAS: Mindful Attention Awareness Scale; RAPM: Raven’s Advanced Progressive Matrices; UMCFAB: University of Miami Computer-Based Functional Assessment Battery.

### Publications of study protocols

Ten of the studies included were protocols, i.e., publications of study methods and planning, but not results^22–38^. Study protocols are regularly published before the intervention is carried out so that its originality and authorship are assured, thus enabling its application by other researchers in different research centers.

Eight of the study protocols^22–29^ had innovative methods and objectives, which, in general, sought to investigate the effects of multiple domain interventions focusing on a range of aspects, such as preventing cognitive impairment, cognitive functions, physical fitness, activities of daily living (ADL), quality of life, gait speed, incidence of falls, and executive functions. For these studies, participants were categorized into CN older adults, elderly at risk for cognitive impairment, subjects with aging-related cognitive impairment, and older patients diagnosed with MCI. Interventions used a variety of resources, such as tablets, computers, physical training (PT), walking, health advice, software such as Fit Brains and Tonic and Phasic Alertness Training (TAPAT), electrostimulation, and vitamins.

Two other studies included in this review were publications of study protocols. Jobe et al.^31^ presented the design of the long-term Advanced Cognitive Training for Independent and Vital Elderly (ACTIVE) study, based on a sample of 2,802 older adults. The participants were randomized into a control group, speed of processing training (SOPT) group, a reasoning training group, and a memory group. The intervention consisted of 10 sessions of 60–75 min over a period of 56 weeks, plus booster sessions for 11 months after the primary training. To determine the long-term effects, assessments were carried out after 1 and 2 years. Similarly, Rebok et al.^10^ applied booster sessions for 35 months after the primary intervention, with data collected 3, 5, and 10 years after the pre-intervention assessment. Due to the magnitude of the ACTIVE study, many studies recruited its data, of which 11 were selected for inclusion in this review.

The study by Kivipelto et al.^30^ described the protocol of the FINGER study, with 1,200 older persons at risk for cognitive impairment. Participants were randomized into two groups. One group received CT combined with nutritional counseling, PT, social activity, and management of metabolic and vascular risk factors, for two 6-month periods, three times a week, totaling 72 sessions of 10–15 min each. The other group consisted of control and was given regular health advice. Long-term effects were to be measured by assessments planned 1 and 2 years after the intervention. One of the studies derived from the FINGER was also included in this review.

### Complete clinical trials

Of the 22 studies whose results were analyzed in this review, 14 involved samples comprising CN older adults, i.e., without MCI and/or early dementia^10,32–44^, whereas 8 involved subjects with MCI or at risk for dementia^6,45–51^.

Ten^10,37–44,51^ of 22 studies drew on data from the ACTIVE study. Besides, a number of studies included employed multimodal interventions, also referred to as multifactorial, namely, FINGER, with nutritional interventions based on a specific diet, physical fitness training programs, and cognitive interventions, such as CT, and also with vascular risk monitoring^6,30^; AgeWell, which includes nutritional counseling, physical activity, CT, optimization of medication, management of vascular risk factors, social activity, and further specific interventions targeting grief and depression^33^; other studies whose interventions include combined CT and PT^22,23,28,32,34,35^; and a study with training of memory, reasoning, problem resolution strategies, visuospatial map reading skills, and handicraft making^34^.

### Objectives

The objectives of the studies varied widely, in which those involving CN subjects tended to investigate the long-term effects of programs for SOPT, reasoning and/or episodic memory on everyday functioning and cognition^10,41,43^, on the trajectory of cognitive aging^42^, increase in cognitive function of older people^33^, impact of CT on objective measures of physical functioning^38^, on use of cognitive strategies^40,44^, on initial recall and learning^37^, on memory for prose^39^, on performance of working memory (WM), and on untrained tasks^36^. Studies with interventions involving combined CT and PT investigated the long-term effects on cognition^32^ and fluid cognitive abilities sensitive to age^35^. Finally, the study by Li et al.^34^ examined the relationship between changes in brain activity and cognitive performance after a multimodal or multifactorial intervention.

The studies with samples comprising older adults with MCI focused on investigating the longitudinal effects of SOPT on processing speed^51^ and examining longitudinal efficacy for cognitive performance of different types of intervention: a program using episodic memory coding strategies^48^, a neuropsychological training (NPT) program in patients treated with cholinesterase inhibitors (ChEIs)^49^, a creative expression therapy (CrExp)^45^, CT plus psychosocial intervention^47^, a computerized cognitive training (CCT) program^46^, and a program of simulated functional tasks (FcTSim)^50^.

With regard to the study in older adults with some cognitive impairment but no MCI diagnosis, the objective was to investigate the longitudinal effects on cognitive functions of a multidomain intervention, combined CT, diet, physical exercise, and cardiovascular risk monitoring^6^.

### Main interventions with cognitively normal older adults

Of the 14 studies conducted in CN samples, 4 studies^10,38,41,42^ analyzed data from the three intervention groups of the ACTIVE study^31^. Based on the protocol of this study, Kwok et al.^33^ conducted a trial in 223 older people with subjective cognitive complaints who received an intervention of 12 sessions of 90 min given weekly, also entailing SOPT, besides memory and reasoning training.

Five studies involving CN elderly were specific interventions for training memory. Three studies analyzed data from memory, training, and control groups in the ACTIVE study, involving a total of 1,401 participants^37,40,44^. The studies of by Gross and Rebok^40^ and Gross et al.^44^ assessed the impact of the memory training program from the ACTIVE study on the use of strategies. In a specific learning intervention involving the memorizing of short stories, the study by Sisco et al.^39^ assessed the impacts of the memory training program from the ACTIVE study in conjunction with the booster intervention, including a total of 1,902 participants^39^. Borella et al.^36^ carried out WM training with 36 older adults who underwent a 2-week intervention of 60-min sessions.

The study by Ball et al.^43^ analyzed the data from the primary SOPT program plus booster sessions of the ACTIVE study, in which 1,400 and 633 older adults participated, respectively, including control groups.

A further two studies in CN older adults applied interventions involving combined CT plus PT^32,35^. The study by Linde et al.^35^, involving 70 senior citizens, applied an intervention comprising weekly 30- to 90-min sessions of PT, CT, and combined PT plus CT, given over a 16-week period. Eggenberger et al.^32^ conducted an intervention with 89 older adults comprising 52 sessions for 1 h, given twice weekly over 26 weeks, consisting of a virtual reality (VR) videogame dancing and treadmill walking with and without simultaneous verbal memory training.

Finally, Li et al.^34^ recruited 270 CN older persons and performed a CT intervention for 12 weeks, twice a week with 1-h sessions, consisting of training of memory, reasoning, problem resolution strategies, visuospatial map reading skills, and production of handcraft.

### Main interventions with older adults with mild cognitive impairment or at risk for dementia

Seven of the eight studies with cognitively impaired individuals examined the effects of a variety of forms of CT in older adults with MCI^45–51^ and one in participants at risk for dementia, but not diagnosed with MCI^6^.

Among the investigations in samples of older subjects with MCI, the study by Valdes et al.^51^ assessed the data of 1,298 participants of the SOPT and control groups of the ACTIVE study. Belleville et al.^47^ carried out an 8-week intervention of eight 2-h sessions. A total of 145 older adults participated in memory training, psychosocial intervention, and a control group. A booster session of the same duration was performed 3 months after the intervention. In the study by Rojas et al.^48^, 46 MCI participants were randomized and the intervention group participated in a 6-month intervention in sessions of 2 h, twice weekly, of CT and cognitive stimulation, including episodic memory training and executive control training techniques. Rozzini et al.^49^ carried out an intervention with 59 participants and 20 sessions of 1 h, five times a week. The investigation groups were divided into a group receiving ChEIs only, a group receiving ChEIs plus NPT (software training memory, language, attention, abstract reasoning, and visuospatial abilities), and a control group. In their study, Bahar-Fuchs et al.^46^ applied an intervention lasting 8–12 weeks with two sessions of 20–30 min/ day, three times a week. Notably, 68 participants were randomized into CCT and active control conditions. In the study by Law et al.^50^, 83 older adults were randomized to receive 13 sessions of functional task exercises (FcTSim) with PT or to an active CT group for 10 weeks. In the study by Zhao et al.^45^, an intervention was performed, comprising 25 sessions of 1 h each over 16 weeks with 93 participants, who were randomized into a CrExp group or a standard CT control group.

With regard to the study in older adults at risk for developing dementia^6^, a multidomain intervention combining PT, diet, cardiovascular risk monitoring, and CT with the use of technology was performed. The authors carried out the intervention based on the protocol of the FINGER study^30^.

### Risk of bias

Regarding the categories of the methodological quality checklist, according to the scores, no articles obtained less than 0.71 points and 23 reached more than 0.80, which corresponds to a high score, excellent quality in the studies analyzed, and low risk of bias. The total average of articles for all categories was 0.84 out of a total of 1.0, meeting the methodological quality requirements of Downs and Black^18^. When divided by domains, the scores achieved were as follows: report 0.84, external validity 0.77, internal validity 0.74, confusion 1.0, and power 0.75 (Table 4).

**Table 4:** Downs and Black’s^18^ checklist results for the present systematic review.

Checklist of Downs and Black ^18^	n	Mean	SD	Minimum	Median	Maximum
Report (converted)	32	0.84	0.04	0.82	0.82	0.91
External validity (converted)	32	0.77	0.32	0.33	1.00	1.00
Internal validity and result bias (converted)	32	0.74	0.07	0.57	0.71	0.86
Confounding factors (converted)	32	1.00	0.00	1.00	1.00	1.00
Power (converted)	32	0.75	0.44	0.00	1.00	1.00
Total (converted)	32	0.84	0.05	0.71	0.86	0.93
Total (original, no conversion)	32	23.41	1.39	20.00	24.00	26.00

SD: standard deviation.

## DISCUSSION

The aim of this study was to carry out a systematic review of studies investigating the long-term effects of CT programs in older adults without dementia. A total of 32 studies were reviewed, comprising 14 in CN older adults, 8 in older adults with MCI and/or at risk of developing dementia, and 10 on study protocols.

### Cognitive training long-term studies with cognitively normal older adults

With regard to the CT long-term studies with CN older adults, and among the studies analyzing data from the three intervention groups of the ACTIVE study, Willis et al.^41^ and Rebok et al.^10^. found less decline in self-reported IADL, particularly in the reasoning training group^41^. Regarding this finding in the functionality of the elderly, Carvalho et al.^52^ highlighted the importance of cognitive interventions, with an emphasis on memory training, to improve the performance on mnemonic tasks.

The results from the studies by Willis et al.^41^ and Rebok et al.^10^. also showed improvements in trained cognitive abilities, with benefits sustained for 5 years after the start of the intervention in all three CT groups^41^ and for 10 years in the reasoning training and SOPT groups.^10^. The study by Rebok et al.^10^ also revealed a medium-to-large effect of the SOPT on processing speed after 10 years. This finding, which represents highly effective maintenance of gains of this type of CT over time, highlights the importance of performing longer follow-up of the effects of CT interventions. In previous year, the study by Ball et al.^43^, also derived from the ACTIVE study, confirmed the maintenance of positive effects of SOPT for 5 years after the intervention, corroborating the results of the study by Willis et al.^41^.

The study by Jones et al.^42^, who drew data from the ACTIVE study, except those related to the booster intervention, showed that memory gains were maintained for 5 years, akin to Willis et al.^41^ and Ball et al.^43^, and that reasoning training significantly attenuated the aging-related limitations in this cognitive ability. The results of the study by Li et al.^34^, included in this review, were consistent with this finding. The authors reported, based on neuroimaging analyses, that CT can promote plastic gains in intrinsic activity patterns, particularly through improvements in functional connectivity and in brain structure which, according to the researchers, are probably part of the neural mechanisms underlying the effects of CT. In other words, according to these findings, CT can slow the pace of cognitive aging.

The results of the study by Ross et al.^38^, however, showed that more training sessions in all intervention groups of the ACTIVE study, i.e., SOPT, memory, and reasoning training, enhanced the performance on tests evaluating fine motor coordination (abilities such as drawing and painting) and gross motor coordination (running, jumping, and walking up and down the stairs), visuomotor coordination (observe, recognize, and use of visual information on shapes, figures, and objects), and also motor speed. However, these results suggested that greater training on reasoning increased hand-grip strength, closely associated with ADL. Effects on cognitive-motor abilities were also observed by Theill et al.^53^, although in this case through simultaneous performance of PT and CT, as opposed to the use of CT alone by Ross et al.^38^. Theill et al.^53^ found that simultaneous training can promote specific improvement in both cognitive performance and dual-task motor-cognitive performance, providing greater potential for performing ADLs.

In a study based on the ACTIVE protocol, Kwok et al.^33^ showed an improvement in general cognitive functioning of low-educated individuals, with effects maintained for at least 9 months in the cognitive areas of conceptualization and memory. The authors proposed this finding might be explained by the ceiling effect, i.e., a tendency of the CT to promote greater gains among subjects with below normal cognition prior to the training and in individuals who received no simultaneous training. In contrast, the results of the study by Teixeira-Fabrício et al.^54^ showed that a higher educational level can lead to greater use of strategies, higher self-efficacy for memory, and larger performance gain post-training. In addition, Casemiro et al.^55^ emphasized that greater education can be directly associated with ease of learning. The researchers reported that high-educated individuals perform visual search tasks more effectively than subjects with a lower educational level.

The studies by Gross and Rebok^40^ and Gross et al.^44^. assessed the impact of the memory training program from the ACTIVE study on the use of strategies. The results of the first of these two studies^40^ indicated that memory training improved the levels of use of strategies and can assist older adults who deploy them in appropriate situations. The authors reported that the effects of training persisted for up to 5 years and that strategies are positively associated with memory performance and daily functioning. The results of the study by Gross et al.^44^ suggested that the method of loci (MoL) (post-training) was used by up to 25% of older adults and immediately improved memory, with effects sustained throughout the follow-up period. These results corroborate the notion that a balance occurs between complexity and novelty in strategy selection by the elderly and that the memory training produces, by promoting changes in the strategies used, observable qualitative and quantitative differences in memory performance. Other studies assessing the effects of memory training on the use of strategies in CN older persons are also available in the literature. In contrast to the findings of Gross and Rebok^40^ and Gross et al.^44^, the results of the study by Yassuda et al.^16^ suggested that the more intense use of memory strategies resulting from training does not necessarily guarantee better performance. Carvalho et al.^52^, however, showed that categorization strategy training led to greater use of the trained strategy and significantly improved the performance on the episodic memory task.

Another study by Gross et al.^37^, also derived from the ACTIVE study, reported an association of memory training with significant long-term gains in learning, stemming from both the highly significant training effect and slower memory decline for up to 5 years. The study by Sisco et al.^9^, which assessed the impacts of the memory training program from the ACTIVE study in conjunction with the booster intervention, suggested in their results that, when carried out in a multifactorial manner together with the booster intervention, the training can improve literal recall for stories.

Borella et al.^36^ showed in their study that WM training produced benefits that were maintained over time. The authors suggested that these findings confirmed there is still room for plasticity in the basic mechanisms of cognition in old age, congruent with other studies addressing CT in CN older adults in which these subjects were able to attain a level of current performance closer to their maximum possible performance^56^.

Ball et al.^43^ reported that positive initial SOPT effects were amplified by booster sessions. According to these authors, a single booster session counteracted around 5 months of age-related processing speed decline. In line with this finding, the results of the study by Aramaki and Yassuda^57^ showed that, besides stability in participants’ cognitive performance between the two interventions, additional gains on episodic memory scales were observed after the booster intervention.

Linde et al.^35^ revealed in the results of their study that the three types of activities carried out by the participants, i.e., PT, CT, and combined PT plus CT, can be seen as cognition-enrichment behaviors. Eggenberger et al.^32^ reported that particular executive functions benefited from simultaneous CT and PT compared to exclusively physical multicomponent training, concluding that cognitive-physical training programs may counteract widespread cognitive impairments in the elderly. These findings are consistent with the recent study by McEwen et al.^58^, who carried out an intervention of simultaneous aerobic exercise and memory training and found that the intervention promoted improvements in memory, attention, and reasoning abilities.

### Long-term studies on cognitive training in older adults with mild cognitive impairment or at risk for dementia

Of the eight studies involving cognitively impaired individuals, seven examined the longitudinal effects of a variety of forms of CT in older adults with MCI^45–51^, while one was a multidomain intervention in elderly people at risk for dementia, but not diagnosed with MCI^6^.

The study by Valdes et al.^51^ revealed that all MCI groups showed an immediate improvement relative to the control group, with an emphasis on the non-amnestic MCI group, in which no significant changes were observed during the 5-year follow-up. Belleville et al.^47^. suggested in their results an improvement on the memory task and strategy use in everyday life of participants of the CT. Consistent with the findings of both studies^47,51^, the study by Olchik^59^, in which older persons with MCI performed memory training, reported that CT can benefit participants in terms of acquisition of strategies for coping with and overcoming cognitive impairment, and even reverse MCI, allowing these individuals to attain a similar level of performance to CN subjects. In addition, the authors believe this training modality represents a cost-effective viable educational intervention that can benefit older persons with MCI.

Rojas et al.^48^ reported that CT in individuals with MCI can also represent a promising treatment option for optimizing performance, preventing cognitive decline, or delaying progression to dementia in this patient group. Brum et al.^60^ noted that CT in older adults with MCI constitutes a non-pharmacological alternative for preventing cognitive and functional decline and for promoting improvement in cognitive performance.

In the study by Rozzini et al.^49^, participants who received ChEIs plus NPT showed significant improvements in cognitive areas and in behavioral disturbances, confirming that a long-term NPT in ChEIs-treated MCI subjects induces additional cognitive and mood benefits. These results are in line with the findings of Olazarán et al.^61^, who reported that patients with MCI, mild Alzheimer’s disease (AD), or moderate AD treated with ChEIs and undergoing a long-term cognitive-motor intervention had greater mood and cognitive benefits compared to the control group.

Bahar-Fuchs et al.^46^ showed in their study that unsupervised home-based CCT with individual tailoring can lead to cognitive and non-cognitive benefits in older adults with MCI. Consistent with these results, the study by Hill et al.^62^ in older adults with MCI and dementia revealed the efficacy of CCT on global cognition, selected cognitive domains, and psychosocial functioning of individuals with MCI.

In the study by Law et al.^50^, the results showed that the FcTSim promoted improvements in general cognitive functions, particularly executive function and problem-solving ability, thereby serving as a cost-effective way of promoting brain plasticity, even in patients with MCI. These findings are consistent with the study by Liao et al.^63^, who randomized older adults with MCI into either a VR-based PT with CT group or a combined PT and CT group without VR. Results showed that the VR group improved global cognition, while both groups improved executive function and verbal memory.

Zhao et al.^45^ suggested in their study that the CrExp therapy promoted greater gains in general cognitive functioning, memory, executive functions, functional status, and everyday living ability among patients receiving the therapy compared to participants receiving standard CT. The authors reported that improvements were maintained at the 6-month follow-up and concluded that this therapy may serve as a cost-effective adjunct to standard interventions for older adults with MCI.

Law et al.^50^ and Zhao et al.^45^ showed that these interventions can serve as cost-effective strategies for older adults with MCI, satisfying the premises of the World Health Organization (WHO), which holds that CT should be provided and applied to both CN older adults and individuals with MCI as a preventive action for cognitive decline and development of dementia, irrespective of social class^64^. This is also guaranteed by the Active Aging policy, which highlights the necessity of incentive for care and development of cognitive abilities to maintain the autonomy of an individual^64^.

With regard to the study in older adults at risk for developing dementia but not diagnosed with MCI^6^, results showed that multidomain intervention can improve cognitive functioning in older adults at risk of cognitive decline^6^.

To sum up, the studies reviewed reported a number of cognitive performance benefits, including a role in improving some motor abilities, among older adults without dementia who participated in CT programs with or without booster sessions and who received multimodal interventions or otherwise.

A total of 14 long-term studies were gathered in which cognitively healthy elderly people, without any type of cognitive impairment, were followed up. In view of this, it was possible to report several cognitive performance benefits. Furthermore, the results of these studies documented that such cognitive benefits lasted up to 5 years after starting the intervention. Eight long-term studies were also gathered in which elderly with MCI or at risk for dementia were followed up. Studies have indicated significant sustained improvements in general cognitive function, executive function, and problem-solving ability, in addition to an increase in brain plasticity. Furthermore, it has also been observed that computerized cognitive interventions at the MCI can prevent cognitive decline or slow conversion to dementia. Finally, 10 publications of protocols were analyzed, studies that will describe their methods and plans. Among them, one protocol has demonstrated the potential to significantly improve efforts to ameliorate cognitive decline, providing important information about the feasibility and intervention effects of a combination of exercise and CT for older adults with MCI.

It is important to highlight that the studies with methodology models included in this review were mostly interventions characterized as multicomponent cognitive stimulation and allow the replication of their methods to other research centers, to verify in a contemporary way to the original authors, if the models of proposed interventions can generate cognitive gains in healthy elderly and in elderly people with MCI.

Therefore, different types of CT programs appear to represent highly applicable cost-effective strategies for promoting health and quality of life in older age. There were a vast number of studies addressing the theme and a wide variety of objectives related to the specific subthemes, with consequent heterogeneity in study results. Generally, however, all findings showed positive effects on the cognition of participants.

The limitations of this study included the selection and inclusion of multimodal CT research; the comparison of CT studies whose participants were CN elderly with studies in which older adults with MCI participated; and the citing of cognitive improvements measured using cognitive screening tests as opposed to more specific tests, such as neuropsychological assessment scales.

As presented in this article, some of the studies employed original, innovative methods incorporating a long-term follow-up. Thus, the methodology of these studies should be replicated in different cultures, given some have been published without results, providing fertile ground for future studies. It is also suggested to carry out future systematic review studies of CT only with a focus on multimodal studies and with samples focused on CN elderly and older adults with MCI.
